# Molecular switch‐mediated detection of *EGFR* deletion mutations and its application to cfDNA analysis

**DOI:** 10.1002/btm2.70105

**Published:** 2025-12-29

**Authors:** Ying‐ying Xu, Lu‐yan Wang, Sheng‐mei Zhou, Hui‐fen Xu, Wang‐yang Pu, Jun‐kang Shen, Chun‐gen Xing, Kai Li, Zhi‐yuan Qian, Li Xiao

**Affiliations:** ^1^ Molecular Medicine Center The Second Affiliated Hospital of Soochow University Suzhou China; ^2^ State Key Laboratory of Radiation Medicine and Protection Soochow University Suzhou China; ^3^ Department of Pharmacy Children's Hospital, Zhejiang University School of Medicine Hangzhou China

**Keywords:** blocker primer, *EGFR* gene, free DNA, molecular switch, mutation

## Abstract

Sensitive and effective detection of epidermal growth factor receptor (*EGFR*) mutations is crucial for the early screening and diagnosis of non‐small cell lung cancer (NSCLC). In this study, we assessed the sensitivity and specificity of the molecular switch technology combined with blocker primers for detecting *EGFR* exon 19 mutations. We demonstrated that this novel method allows real‐time detection of mutated templates on a qPCR platform. Moreover, applying this method to cell‐free DNA samples enhances the mutation detection rate.

**Translational Impact Statement**

In this study, we demonstrated that a molecular switch technology combined with blocker primers can detect the mutation of exon 19 in *EGFR* in real‐time on a qPCR platform, and improve the detection sensitivity of low copy DNA samples. This advancement holds promise for enhancing early lung cancer diagnosis, promoting efficient non‐invasive testing methods, and enabling personalized treatment strategies.

## 1 INTRODUCTION

Epidermal growth factor receptor (*EGFR*) mutations are prevalent among patients with non‐small cell lung cancer (NSCLC) and constitute a major focus of clinical research.^1^, ^2^, ^3^ Tanaka et al. identified that in Orientals, the del E746‐A750 (15 bp deletion) and del L747‐S752 ins S (18 bp deletion) mutations in *EGFR* exon 19 constitute approximately 68% of cases.^4^, ^5^ Detection of these mutations is crucial for early tumor screening and prognostic assessment in NSCLC patients.

Currently, mutation detection in tumor tissues typically relies on DNA samples derived from somatic cells. However, the low copy numbers of mutations in somatic cells can sometimes be obscured by the abundance of wild‐type (WT) genes in samples, posing challenges for detection.^6^, ^7^, ^8^ Although gene sequencing is the gold standard, it is limited by its low sensitivity, time‐consuming, and high cost, thereby restricting its clinical utility, particularly in the case of circulating free DNA samples.^9^, ^10^

In recent years, liquid biopsy technology, exemplified by the detection of circulating tumor DNA (ctDNA) in blood samples, has gained increasing prominence in cancer detection due to its non‐invasiveness, ease of operation, and ability for repeated sampling.^11^, ^12^, ^13^, ^14^ *EGFR* gene mutations present in primary or metastatic lesions of lung cancer patients can be detected in their circulating free DNA.^15^ With respect to clinical diagnosis, high sensitivity and specificity could be achieved for cfDNA samples when various optical molecular biology techniques are applied, which holds promising applications in early diagnosis, personalized treatment, and prognosis of lung cancer.^16^, ^17^, ^18^ However, early‐stage tumor patients often have low levels of circulating free tumor DNA in their blood,^19^ and current detection technologies may not effectively detect mutated ctDNA.^20^, ^21^ Establishing new detection methods with high sensitivity and specificity for mutated genes is urgently needed to monitor mutation levels in lung cancer patients.

**FIGURE 1:**
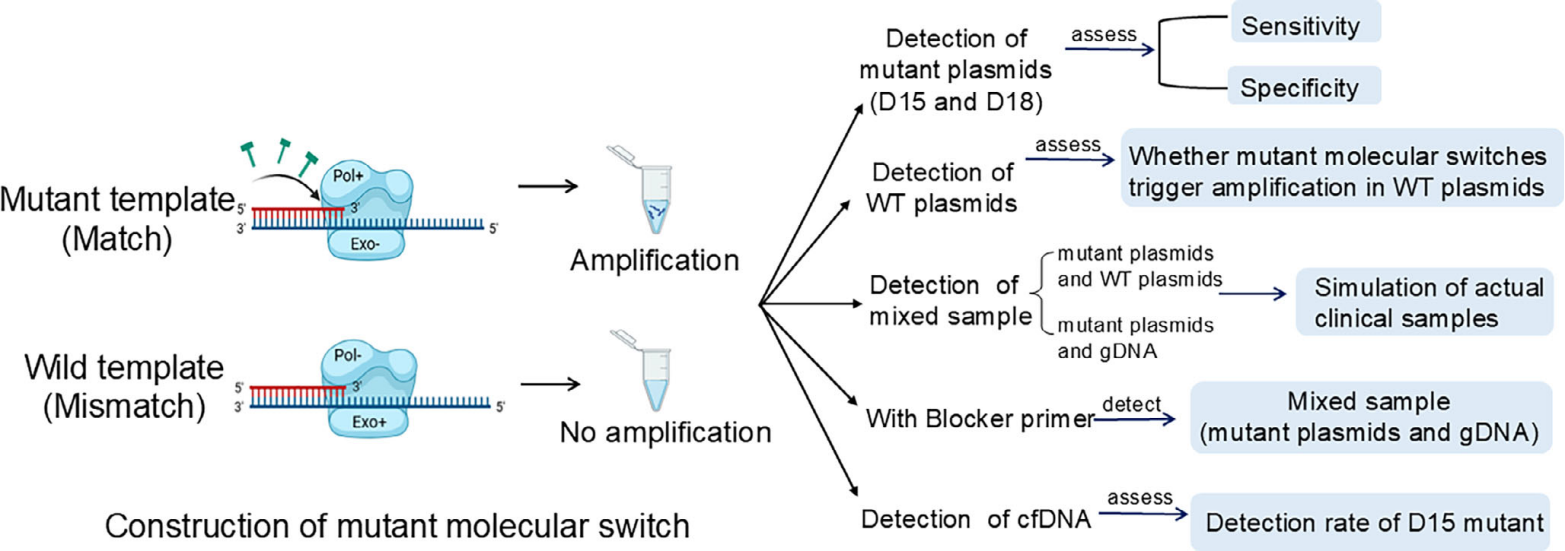
Flowchart of the experiment.

This paper evaluated the sensitivity and specificity of molecular switch assay in detecting *EGFR* exon 19 del E746‐A750 (15 bp deletion) and del L747‐S752 ins S (18 bp deletion). The strategy of molecular switch assay combined exonuclease‐resistant phosphorothioate‐modified base‐specific primers with a high‐fidelity DNA polymerase possessing 3′ → 5′ exonuclease activity. The polymerization center and the 3′ → 5′ exonuclease hydrolysis center in the high‐fidelity polymerase molecule functioned in an “on” and “off” manner in molecular switch: the high‐fidelity DNA polymerization directly occurred at the enzyme's polymerization center for paired primers. In contrast, for the 3′‐terminal mismatch primer, it transferred from the enzyme's polymerization center to the 3′ → 5′ exonuclease digestion center. Due to the exonuclease‐resistant characteristics of the modified 3′‐terminal, this resulted in a prolonged digestion process without product, ultimately halting the DNA polymerization reaction due to the enzyme's polymerization center becoming inactive.^22^, ^23^ In this study, two hotspot deletions in *EGFR* exon 19 were chosen as the detection targets, and primers were designed to specifically pair with the mutant loci at their 3′ ends, incorporating phosphate modifications. The molecular switch assay enabled amplification of the mutant sequence when primers matched the mutant template, while amplification was inhibited when primers mismatched with the wild‐type template. This technique was employed to establish a highly sensitive and specific qPCR assay for detecting deletion mutations at bases 15 and 18 of exon 19 on a real‐time qPCR platform. To enhance sensitivity and reduce non‐specific amplification, blocker primers^24^, ^25^ were further introduced into the molecular switch assay. These oligonucleotides were fully complementary to the wild‐type sequence in the mutated region being targeted and modified at the 3′ end to block extension. By binding to the wild‐type template, Blocker primers prevented amplification of the wild‐type sequence, thereby facilitating amplification and enrichment of the target mutant fragments. This incorporation of blocker primers optimized the molecular switch for detecting samples with low mutant copies. Furthermore, the molecular switch utilized in this study demonstrated applicability for mutation detection in free DNA samples characterized by low copy numbers. This approach offers potential for non‐invasive diagnosis of exon 19 deletion mutations in the *EGFR* gene among lung cancer patients and personalized medication monitoring. The experimental workflow is illustrated in Figure 1.

## 2 MATERIALS AND METHODS

The *E. coli* DH5α strain was purchased from Beijing Tiangen, plasmid PMD‐19 from TaKaRa (Dalian, China), and pGEM‐T simple vector from Promega (Madison, USA). Genomic DNA extraction kits for blood/cell/tissue were obtained from Beijing Tiangen, while QIAamp circulating nucleic acid kit, QIAquick gel extraction kit, MinElute PCR purification kit, and QIAprep plasmid mini‐extraction kit were from QIAGEN. The BigDye Terminator sequencing kit 3.1 was sourced from Applied Biosystems, and HotStarTaq Plus DNA Polymerase and dNTP Mix from Qiagen. BIOMIGA provided the 2× Taq PCR Mix, and Biotiu supplied the 20× Eva green. Thermo Scientific products included high‐fidelity DNA polymerase, T4 ligase, 100 bp DNA Ladder, 1 kb DNA Ladder, and restriction endonucleases SalI and XbaI. Additional materials such as ampicillin, Tris‐base, EDTA, DMSO, electrophoretic grade agarose, yeast extract, tryptone, and agar powder were primarily sourced from Alpha Bio Reagent.

The primers utilized in this study were synthesized by Shanghai Biotechnology Company, China. These included three types: primers for wild‐type template preparation, fixed‐point mutation primers, and molecular switch detection primers.

### 2.1 Construction of both wild and mutant recombinant plasmids

The plasmids containing both WT and mutant *EGFR* genes were constructed using conventional PCR and overlapping extension fixed‐point mutagenesis with Taq DNA polymerase, employing the primers detailed in Table 1. PCR products were isolated and purified via agarose gel electrophoresis using the MinElute PCR purification kit. Gel‐purified PCR products were subcloned into the pGEM‐T easy vector using T4 DNA Ligase. Subsequently, the cloned products were sequenced to confirm their fidelity.

**TABLE 1:** Primer sequences for constructing wild and mutant plasmids.

Primer name	Sequence
Primer 1	5′‐TGTGATTCGTGGAGCCCAACA‐3′
Primer 2	5′‐AGGCCAGTGCTGTCTCTAAG‐3′
Primer 3	5′‐GGCTTTCGGAGATGTTTTGATAGCGACGGGAATTTTAACTTTCTC‐3′
Primer 4	5′‐GCTATCAAAACATCTCCGAAAGCCAACAAGGAA ATCCTCGAT‐3′
Primer 5	5′‐CTTGTTGGCTTTCGATTCCTTGATAGCGACGGGAATTTTAAC‐3′
Primer 6	5′‐ATCAAGGAATCGAAAGCCAACAAGGAAATCCTCGAT‐3′

*Note*: Primers 1 and 2 were used for constructing the WT recombinant plasmid; Primers 1, 2, 3, and 4 were used for constructing the D15 mutant recombinant plasmid; Primers 1, 2, 5, and 6 were used for constructing the D18 mutant recombinant plasmid.

### 2.2 Genomic DNA samples and cfDNA sample preparation

Two normal human blood samples were obtained from the Second Affiliated Hospital of Soochow University. Genomic DNA was extracted from these blood samples following the protocol of the Blood/Cell/Tissue Genomic DNA Extraction Kit.

Surgical tissue samples and peripheral blood samples were collected from 24 lung cancer patients at the Second Affiliated Hospital of Soochow University and Shanghai Lung Hospital. Genomic DNA from tissue samples and cfDNA from peripheral blood were extracted using the Blood/Cell/Tissue Genomic DNA Extraction Kit and the QIAamp Circulating Nucleic Acid Kit, respectively.

### 2.3 Construction of a molecular switch detection system

The molecular switch assay employed high‐fidelity DNA polymerase in conjunction with specific primers featuring 3′ end thio‐modification. Table 2 details the primers utilized in this study for detecting the D15 and D18 mutants using the molecular switch. Primer 7 and Primer 8 served as forward primers with 3′ end thio‐modification, while Primer 9 functioned as a reverse primer without specific modification.

**TABLE 2:** Molecular switch primer sequences for detection of mutant templates.

Primer name	Sequence
Primer 7	5′‐TTCCCGTCGCTATCAAAAG‐3′
Primer 8	5′‐CGTCGCTATCAAGGAATCGT‐3′
Primer 9	5′‐AGGCCAGTGCTGTCTCTAAG‐3′

*Note*: Primers 7 and 9 were used for detecting mutations in the EGFR gene type D15 with the molecular switch; Primers 8 and 9 were used for detecting mutations in the EGFR gene type D18 with the molecular switch. Underlined bases denote sulfide modifications.

Blocker primers were incorporated into the PCR reaction system of the molecular switch in equimolar amounts relative to the forward or reverse primers. Subsequently, qPCR reactions were conducted.

### 2.4 Ethics statement

This study was registered at the Medical Ethics Committee of the second affiliated hospital of Soochow university prior to inclusion (EC‐JD20240601). All patients in this study gave written consent, and all relevant investigations were performed according to the principles of the declaration of Helsinki.

## 3 RESULTS

### 3.1 Successful construction of the recombinant plasmids

**FIGURE 2:**
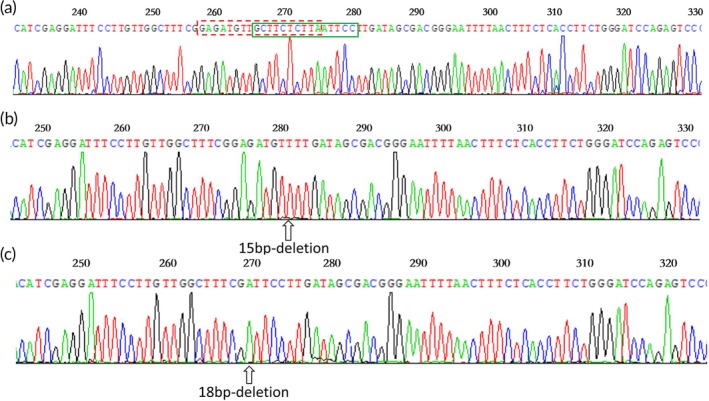
Recombinant plasmid sequencing analysis results. (a) Sequencing results of WT plasmid, with the mutant site indicated by the rectangular box. (b) Sequencing results of D15 mutant plasmid. (c) Sequencing results of D18 mutant plasmid.

The WT and mutant plasmid templates were successfully prepared, as depicted in Figure 2. Sequencing confirmed that the subcloned vectors contained both WT and mutant fragments. Specifically, the WT recombinant plasmid encompassed the exon 19 sequence of the *EGFR* gene (Figure 2a), the D15 mutant plasmid harbored the *EGFR* gene exon 19 del E746‐A750 deletion mutation sequence (Figure 2b), and the D18 mutant plasmid contained the sequence of the *EGFR* gene exon 19 del L747‐S752 ins S deletion mutation (Figure 2c).

### 3.2 Sensitivity and specificity of the molecular switch for detecting D15 and D18 mutant templates

**FIGURE 3:**
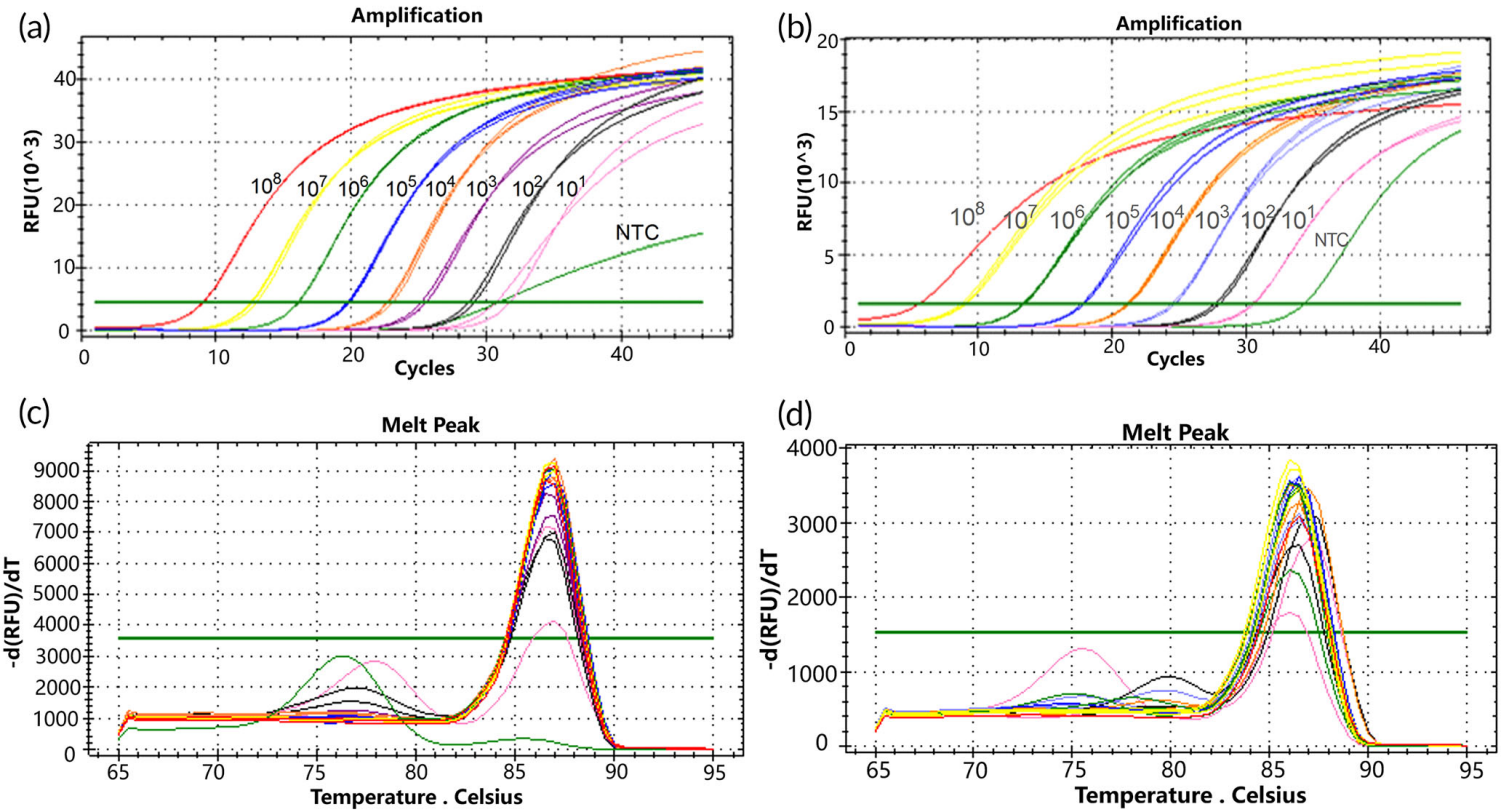
qPCR amplification curves of 10‐fold isobaric concentration gradients for molecular switch detection of mutant plasmids (a) D15 mutant. (b) D18 mutant. qPCR melting curves of 10‐fold isobaric concentration gradients for molecular switch detection of mutant plasmids (c) D15 mutant. (d) D18 mutant.

A 10‐fold isobaric gradient dilution of D15 and D18 mutant plasmids was conducted, starting with a plasmid concentration of 10^8^ copies/μL, with each dilution replicated in three parallel tubes. Real‐time qPCR amplification of the two mutant templates was performed using the mutant molecular switch system, ranging from 10^8^ to 10^1^ copies/μL. The sensitivity of the molecular switch system for detecting D15 and D18 mutations was assessed based on qPCR amplification curves and melting curves. The molecular switch effectively amplified both D15‐type (Figure 3a) and D18‐type (Figure 3b) mutant plasmids down to a minimum concentration of 10^1^ copies/μL. To test the specificity of the molecular switch for mutant templates, melting curves were analyzed. The melting curves demonstrated a single peak at copy concentrations ranging from 10^8^ to 10^2^ copies/μL for the D15‐type mutant plasmid, indicating good specificity. However, at the lowest concentration of 10^1^ copies/μL, non‐specific band amplification was observed (Figure 3c). Similarly, the molecular switch exhibited a single peak at copy concentrations ranging from 10^8^ to 10^3^ copies/μL for the D18‐type mutant plasmid, also showing good specificity. Nevertheless, at concentrations of 10^2^ and 10^1^ copies/μL, non‐specific band amplification occurred (Figure 3d).

**FIGURE 4:**
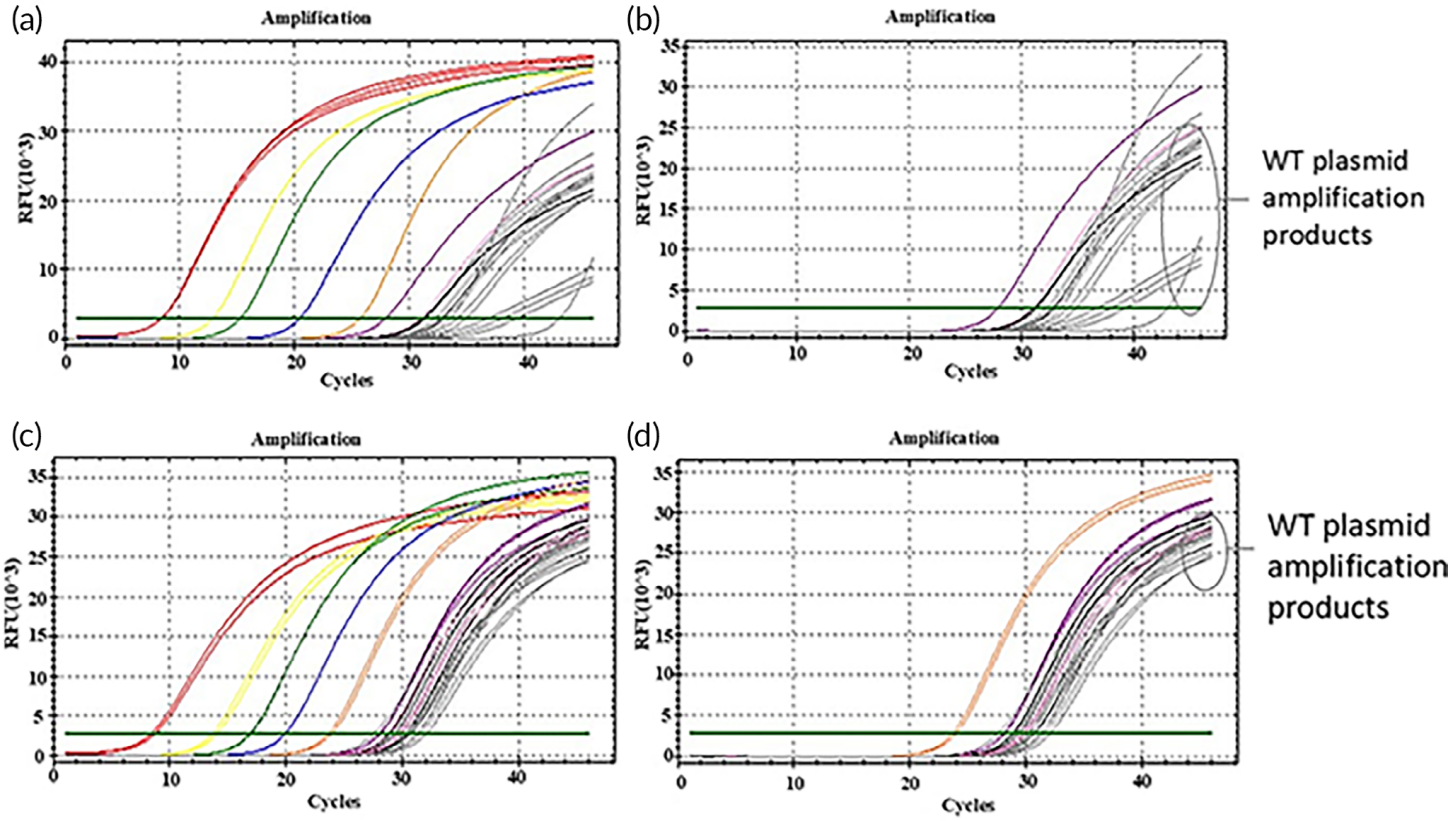
Amplification curves of mutant plasmids and WT plasmids detected by mutant molecular switches. (a) Amplification curves of D15 mutant molecular switch at concentrations of 10^8^ to 10^1^ copies/μL for the D15 mutant plasmid and 10^5^ to 10^1^ copies/μL for the WT plasmid. (b) Amplification curves of D15 mutant molecular switch at concentrations of 10^3^ to 10^1^ copies/μL for the D15 mutant plasmid and 10^5^ to 10^1^ copies/μL for the WT plasmid. (c) Amplification curves of D18 mutant molecular switch at concentrations of 10^8^ to 10^1^ copies/μL for the D18 mutant plasmid and 10^5^ to 10^1^ copies/μL for the WT plasmid. (d) Amplification curves of D18 mutant molecular switch at concentrations of 10^4^ to 10^1^ copies/μL for the D18 mutant plasmid and 10^5^ to 10^1^ copies/μL for the WT plasmid.

**FIGURE 5:**
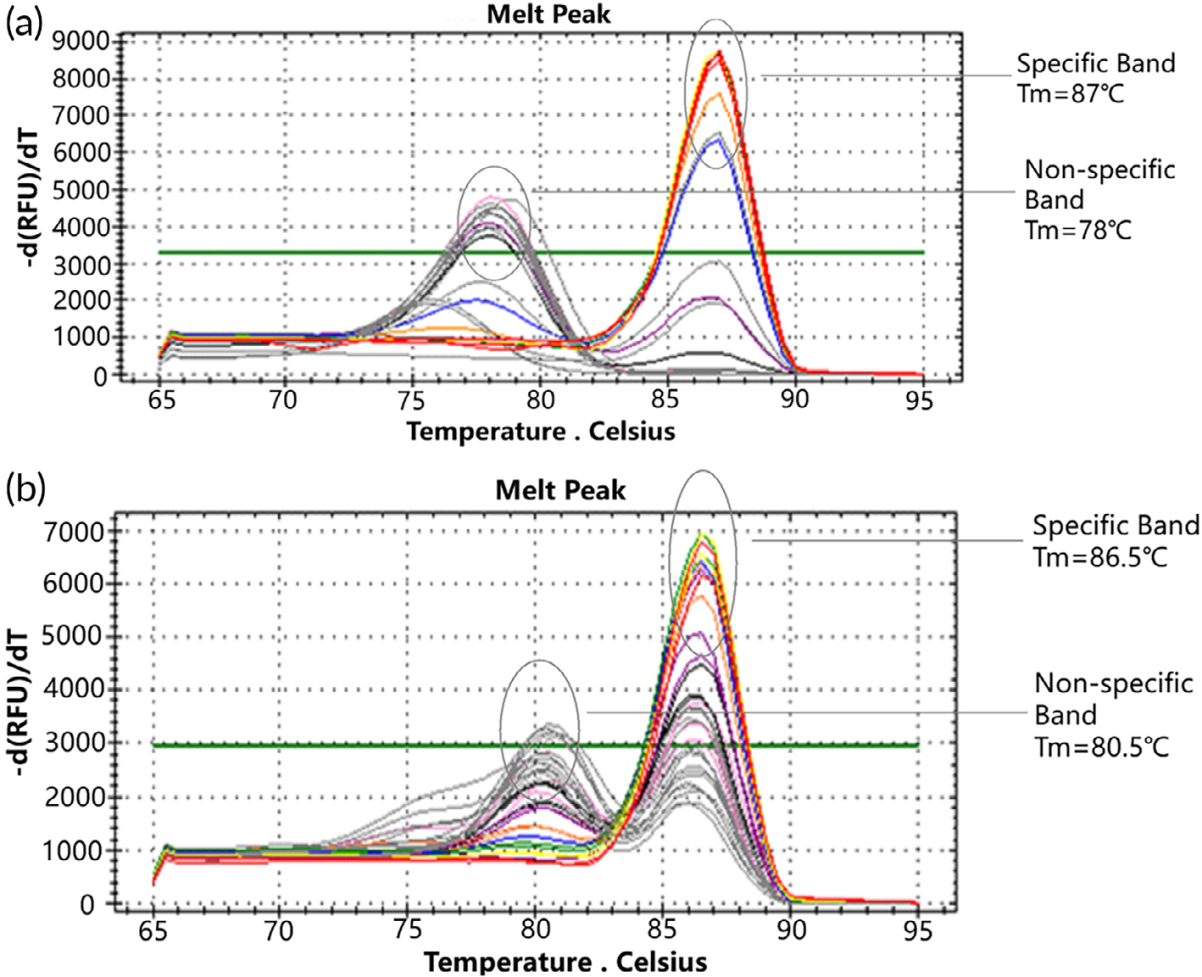
Melting curves of the molecular switch in the mutant plasmid (concentration of 10^8^ to 10^1^ copies/μL) and in the WT plasmid (concentration of 10^5^ to 10^1^ copies/μL). (a) D15 type. (b) D18 type.

A 10‐fold isocratic dilution of the WT plasmid was performed, starting with a plasmid concentration of 10^5^ copies/μL, with each dilution repeated in three parallel tubes. Real‐time qPCR amplification of wild‐type templates ranging from 10^5^ to 10^1^ copies/μL utilized the D15 and D18 mutant molecular switch systems. The amplification efficiency of these mutant molecular switches in the WT plasmid assay was assessed through qPCR amplification curves, melting curves, and Ct values. Both the D15 and D18 mutant molecular switches exhibited incomplete qPCR amplification profiles in WT plasmids ranging from 10^5^ to 10^1^ copies/μL, with amplification occurring after 33 and 29 cycles, respectively (Figure 4). Furthermore, the standard deviation of the Ct values exceeded 0.25, indicating poor accuracy (Table 3). Melting curve analysis revealed that the D15 mutant molecular switch displayed double peaks in one tube each at WT plasmid concentrations of 10^5^ copies/μL and 10^3^ copies/μL, with a product having a Tm of 87°C, showing a low peak and fluorescence value below the green threshold line, while other WT plasmid copy numbers showed non‐specific amplification products (Tm = 78°C) (Figure 5a). The D18 mutant molecular switch exhibited double peaks in one tube each at WT plasmid concentrations of 10^4^ and 10^2^ copies/μL, and in two tubes at 10^3^ copies/μL, yielding a product with a Tm of 86.5°C, whereas the remaining WT plasmid copies showed non‐specific amplification products (Tm = 80.5°C) (Figure 5b). These results indicate that the D15 mutant molecular switch exhibits high specificity in qPCR assays at WT plasmid concentrations of 10^5^ to 10^1^ copies/μL and is not prone to false‐positive results, whereas the D18 mutant molecular switch shows slightly lower specificity in this concentration range and may generate false‐positive results.

**TABLE 3:** Ct values for amplification of the deletion mutant molecular switch in the mutant plasmid and the WT plasmid.

Sample	Starting quantity (SQ)	Ct mean	Sample	Starting quantity (SQ)	Ct mean
D15	10^8^	8.52 ± 0.017	D18	10^8^	8.64 ± 0.253
	10^7^	13.45 ± 0.617		10^7^	13.93 ± 0.293
	10^6^	20.51 ± 0.251		10^6^	17.02 ± 0.039
	10^5^	21.08 ± 0.755		10^5^	19.60 ± 0.597
	10^4^	25.14 ± 1.007		10^4^	23.85 ± 0.254
	10^3^	31.52 ± 0.119		10^3^	28.15 ± 0.019
	10^2^	30.07 ± 1.727		10^2^	29.60 ± 0.728
	10^1^	29.97 ± 1.508		10^1^	29.50 ± 0.599
WT15	10^5^	33.03 ± 1.828	WT18	10^5^	31.31 ± 1.015
	10^4^	36.20 ± 2.760		10^4^	29.40 ± 1.573
	10^3^	33.17 ± 0.451		10^3^	30.75 ± 0.532
	10^2^	35.16 ± 4.094		10^2^	30.98 ± 0.293
	10^1^	35.56 ± 5.767		10^1^	29.72 ± 0.768
NTC		31.31	NTC		30.82

**FIGURE 6:**
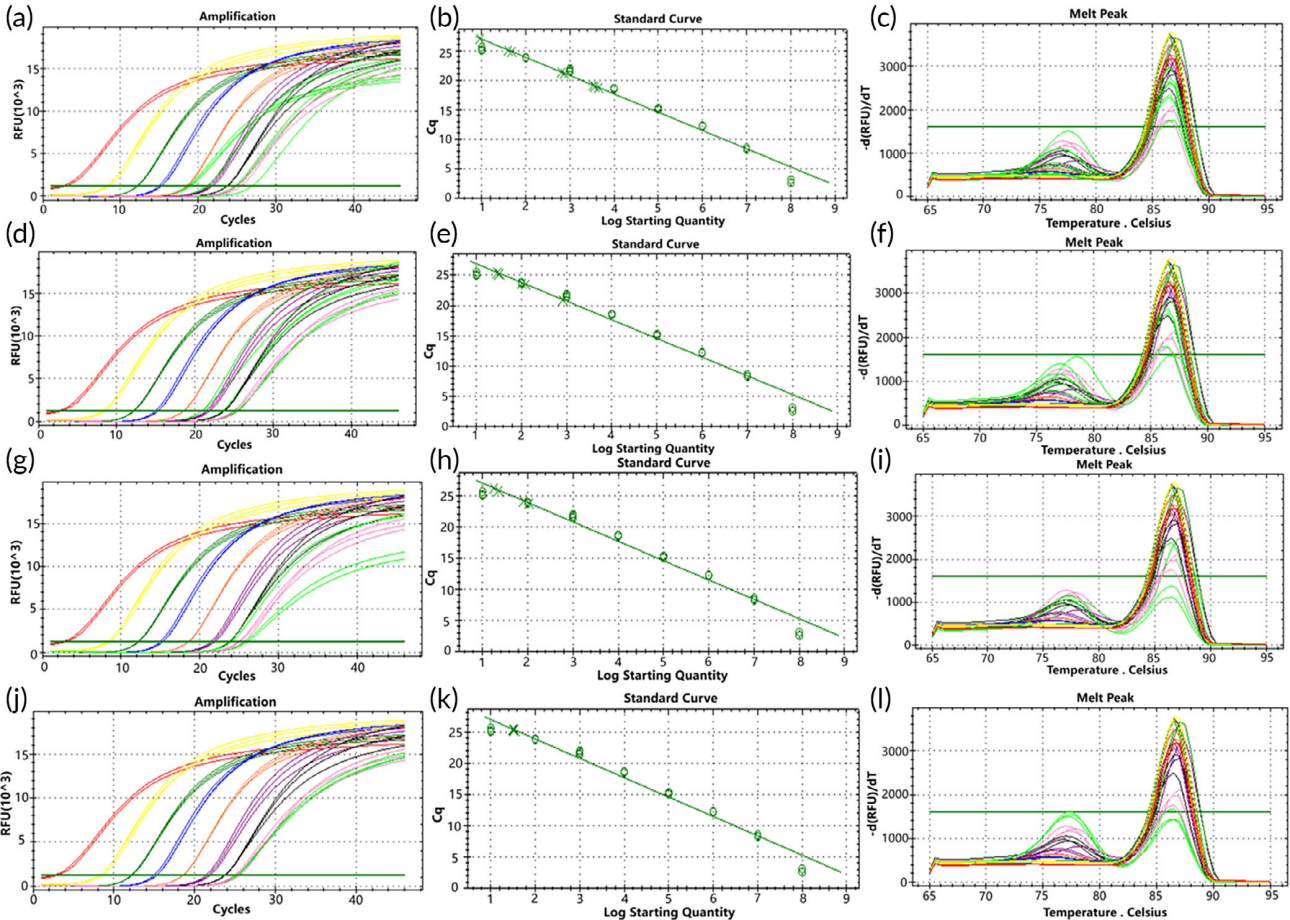
Standard curve of D15 mutant molecular switch, showing amplification curves and melting curves of mixed samples detected by molecular switches in gDNA backgrounds with different copy numbers (green represents the amplification curve of mixed plasmid templates). Panels (a) to (c) depict detection of mixed mutant plasmid (concentration of 10^4^ to 10^1^ copies/μL) in a gDNA background of 10^4^ copies/μL; panels (d) to (f) show detection of mixed mutant plasmid (concentration of 10^3^ to 10^1^ copies/μL) in a gDNA background of 10^3^ copies/μL; panels (g) to (i) illustrate detection of mixed mutant plasmid (concentration of 10^2^ to 10^1^ copies/μL) in a gDNA background of 10^2^ copies/μL; panels (j) to (l) show the mixed concentration of 10^1^ copies/μL mutant plasmid detected in a gDNA background of 10^1^ copies/μL.

**FIGURE 7:**
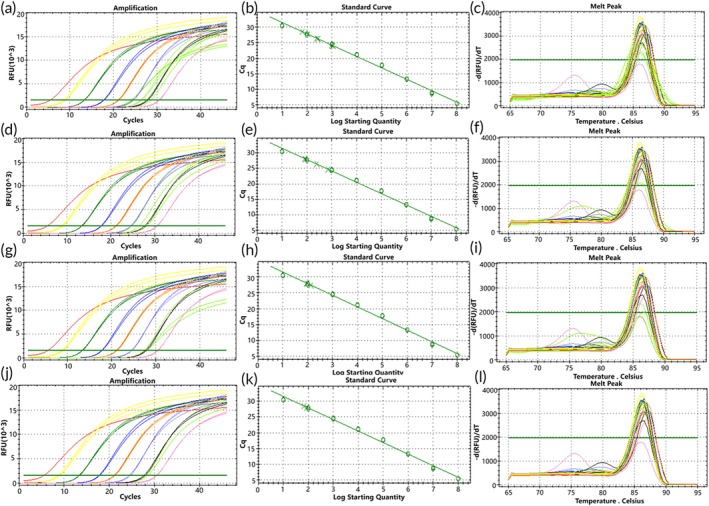
D18 mutant molecular switch standard curve, showing amplification curves and melting curves of mixed samples detected by molecular switches in gDNA with different copy number backgrounds (green represents the amplification curve of mixed plasmid templates). Panels (a) to (c) depict detection of mixed mutant plasmid (concentration of 10^4^ to 10^1^ copies/μL) in a gDNA background of 10^4^ copies/μL; panels (d) to (f) show detection of mixed mutant plasmid (concentration of 10^3^ to 10^1^ copies/μL) in a gDNA background of 10^3^ copies/μL; panels (g) to (i) illustrate detection of mixed mutant plasmid (concentration of 10^2^ to 10^1^ copies/μL) in a gDNA background of 10^2^ copies/μL; panels (j) to (l) show the mixed concentration of 10^1^ copies/μL mutant plasmid detected in a gDNA background of 10^1^ copies/μL.

To simulate a molecular environment closer to the two types of *EGFR* gene rare deletion mutations found in actual clinical samples, genomic DNA (gDNA) was mixed with mutation templates in different ratios. When gDNA was mixed with the two types of mutant plasmids, the amplification curves of both D15 and D18 mutant plasmids showed more consistency with the standard curves at a gDNA background of 10^4^ to 10^1^ copies/μL. Predominantly specific product peaks were observed at high template concentrations, while low‐copy mutant plasmids exhibited bimodal peaks with nonspecific products (Figures 6 and 7).

**FIGURE 8:**
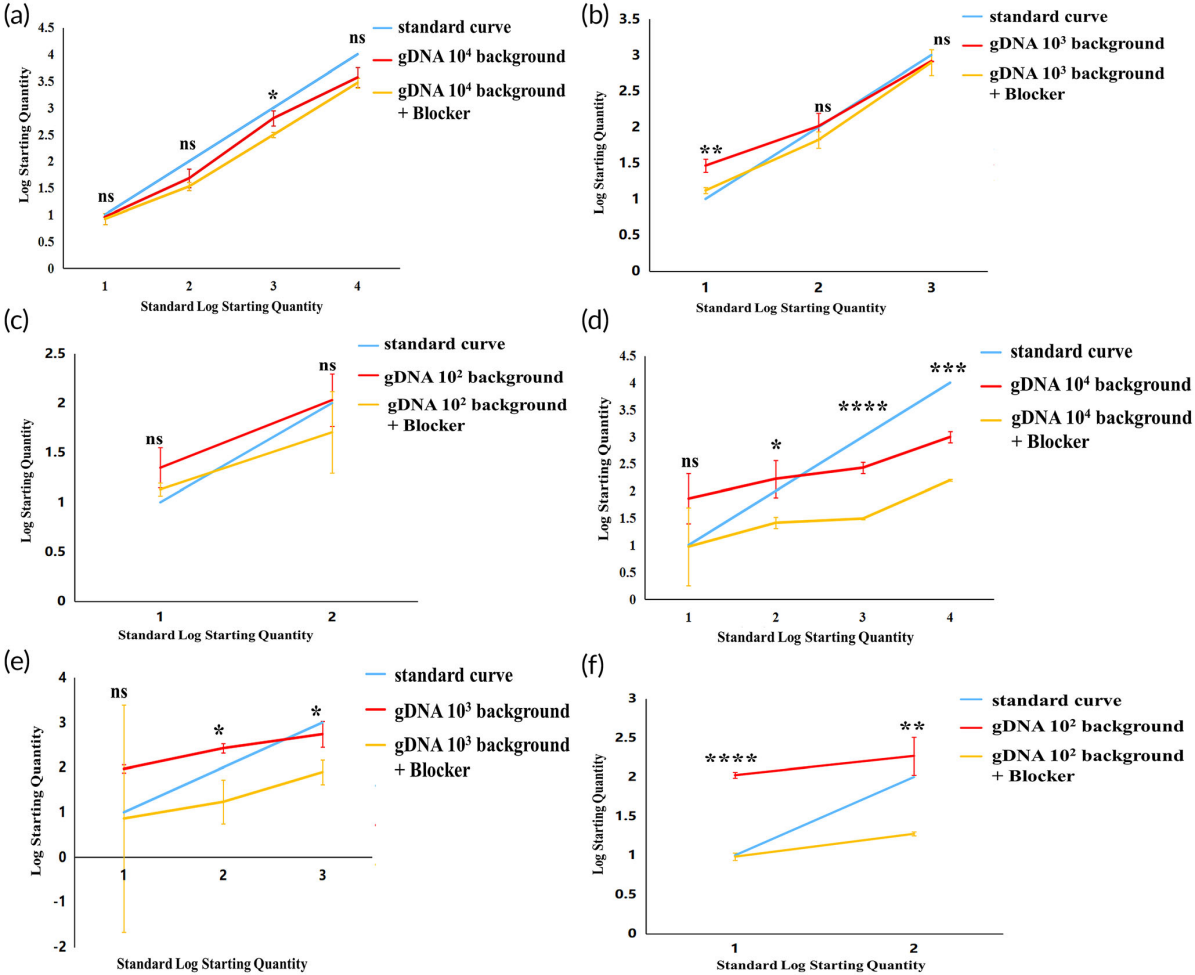
Statistical plots comparing the correlation of molecular switch incorporation of Blocker primers for detecting mutation models in different copy number backgrounds of gDNA, compared with systems without Blocker primers and standard curves. Panels (a) to (c) depict D15 type; panels (d) to (f) depict D18 type.

Statistical analysis of these results indicated strong correlation of the molecular switch with the standard curve for both types of mutations detected in gDNA backgrounds of 10^4^ and 10^3^ copies/μL (Figure 8a,b,d,e), whereas the correlation was poor for both types of mutations detected in a gDNA background of 10^2^ copies/μL (Figure 8c,f). Additionally, the D15‐type mutation showed stronger correlation with the standard curve compared to the D18‐type mutation across all mentioned backgrounds. These results suggest that the mutant molecular switch exhibits good sensitivity in detecting D15‐type mutations in genomic DNA backgrounds with a minimum of 10^1^ copies/μL of mutant molecules. However, sensitivity for detecting D18‐type mutations is slightly lower, indicating a need for further optimization to improve detection sensitivity.

To optimize the molecular switch technology, we retested a mixed template consisting of gDNA and mutant plasmid after adding the Blocker primer to its PCR system. The study revealed that at a gDNA background of 10^4^ copies/μL (Figure 8a), the D15 mutant molecular switch with Blocker primer added exhibited poorer correlation with the standard curve compared to the system without Blocker primer; When the Standard Log Starting Quantity was 3, the difference between the assay results before and after the addition of Blocker primers was statistically significant (*p* < 0.05). Similarly, the D18 type showed weaker correlation with the standard curve when detecting mutant systems ranging from 10^4^ to 10^2^ copies/μL (Figure 8d), but stronger correlation when detecting the mutant system at 10^1^ copies/μL; When the Standard Log Starting Quantity was 2 to 4, the differences between the assay results before and after the addition of Blocker primers were statistically significant (*p* < 0.05). At gDNA backgrounds of 10^3^ and 10^2^ copies/μL (Figure 8b,c,e,f), both mutant molecular switches demonstrated improved correlation with the standard curves in samples with low copy numbers of mutant molecules when the Blocker primer system was employed, in contrast to when no Blocker primer was used; The D15 mutation molecular switch only showed statistically significant differences in detection results before and after adding Blocker primers when gDNA background of 10^4^ copies/μL and Standard Log Starting Quantity was 1 (*p* < 0.05), while the D18 type showed the opposite, with no statistically significant differences in detection results produced only under the above conditions (*p* > 0.05). These findings indicate that incorporating Blocker primers into the D15 and D18 mutant molecular switches enhances the detection of mutations in backgrounds with low‐copy templates and mutant molecules. This approach is particularly effective for detecting samples with low template background and mutant copy numbers.

### 3.3 Molecular switches were tested for plasma free DNA samples on the Real‐time qPCR platform

**FIGURE 9:**
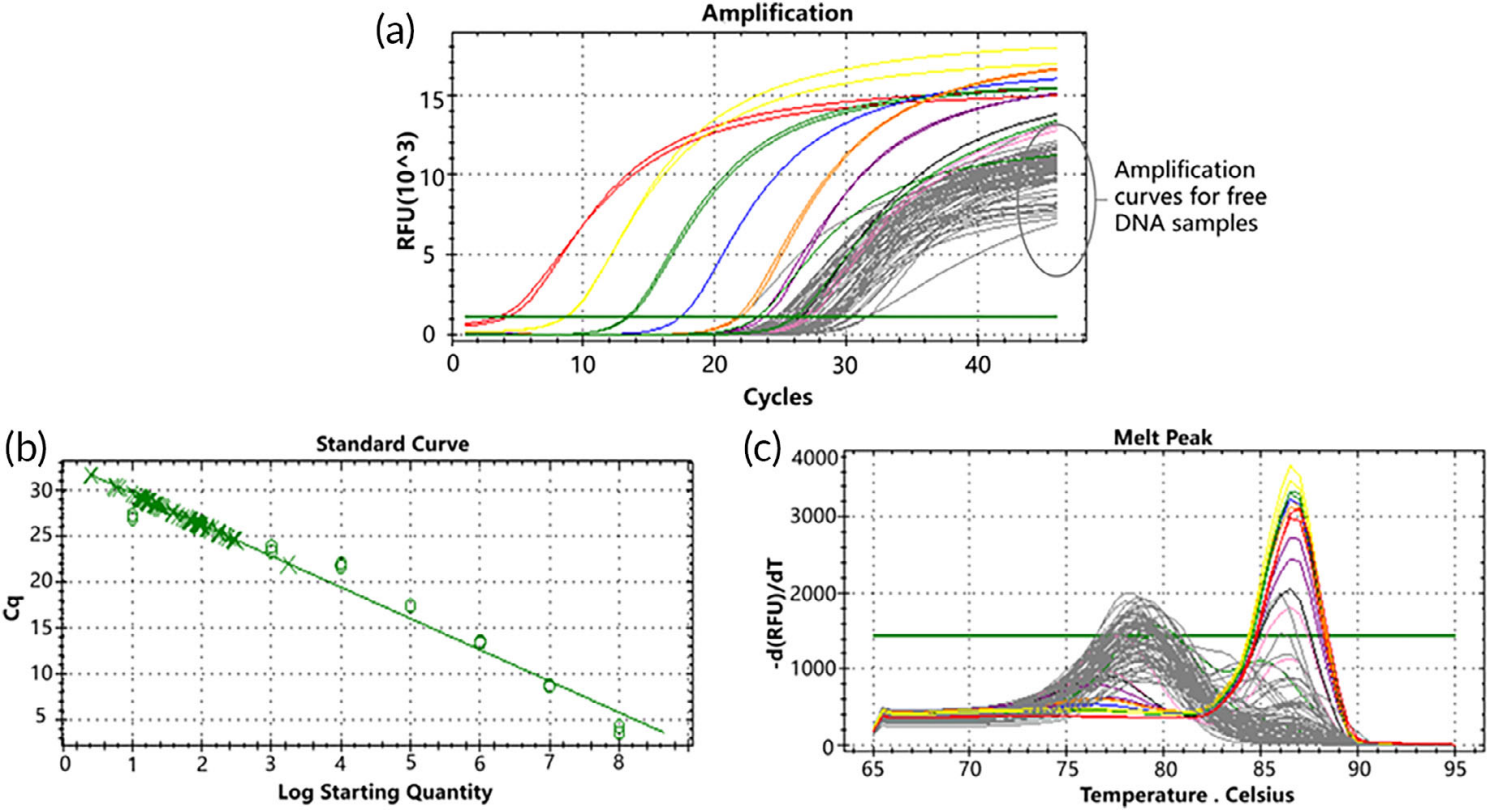
qPCR amplification curve, standard curve, and melting curve of D15 mutant molecular switch in 24 cases of free DNA.

Peripheral blood samples were collected from 24 lung cancer patients, and plasma free DNA was extracted. The free DNA samples were then analyzed using the D15 mutant molecular switch technique. The detection rate of the D15 mutant in plasma free DNA samples was assessed using the standard curve of the D15 mutant molecular switch as a control in the molecular switch system. The study observed that the Ct values of qPCR amplification of free DNA from the 24 lung cancer patients ranged from 24.96 to 29.67, indicating starting copy numbers ranging from 100.998 to 102.387 according to the standard curve. Melting curve analysis revealed that some free DNA samples exhibited double peaks (Tm = 87°C), while others showed single peaks (Tm = 78°C) (Figure 9). This suggests the detection of *EGFR* gene exon 19 D15 mutations in the 24 free DNA samples.

**FIGURE 10:**
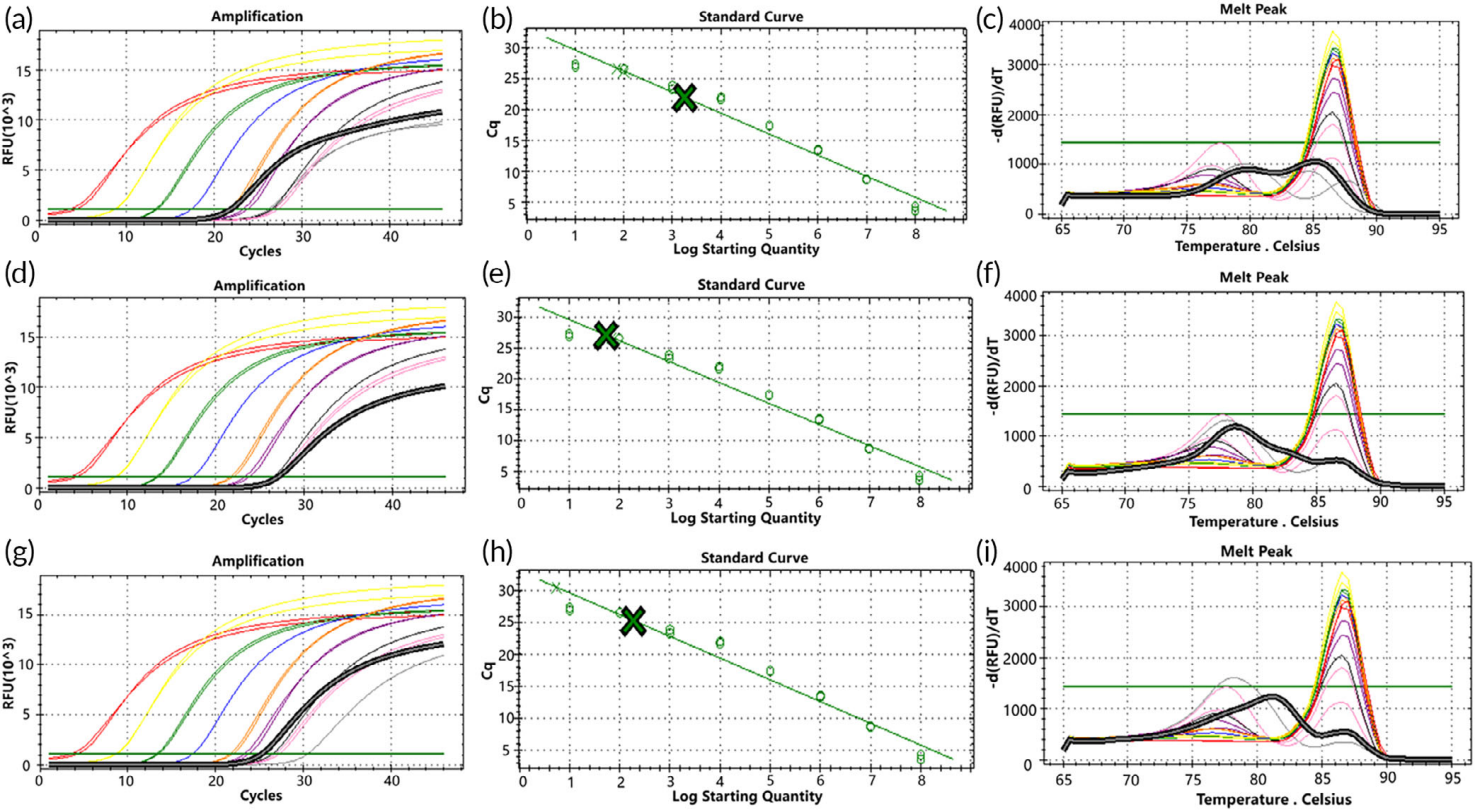
qPCR amplification curves, standard curves, and melting curves of D15 mutant molecular switches in three free DNA samples. Panels (a) to (c) depict free DNA samples No. 7; panels (d) to (f) depict free DNA samples No. 23; panels (g) to (i) depict free DNA samples No. 24.

Figure 10 depicts amplification curve graphs, melting curve graphs, and comparisons with the standard curve for three cases of free DNA samples where the mutation in exon 19 (type D15) of the *EGFR* gene was detected. The lowest Ct value was observed in the amplification of free DNA sample No. 7, with a calculated starting copy number of 103.248 according to the standard curve. Melting curve analysis revealed a qPCR product with a bimodal peak, consisting of specific and non‐specific bands.

**FIGURE 11:**
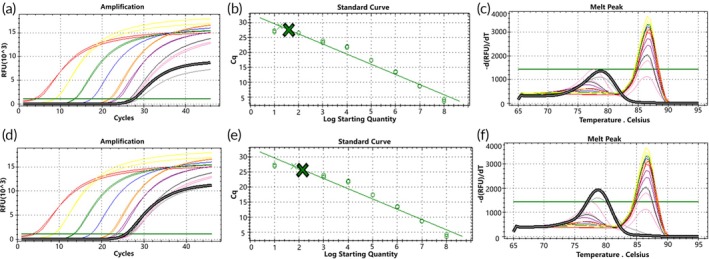
qPCR amplification curves, standard curves, and melting curves of D15 mutant molecular switches in two free DNA samples with no detectable D15 mutation. Panels (a) to (c) depict free DNA samples No. 1; panels (d) to (f) depict free DNA samples No. 4.

Figure 11 presents amplification curve graphs, melting curve graphs, and comparisons with the standard curve for two cases of free DNA samples that did not detect the *EGFR* gene exon 19 D15 type mutation. The amplification melting curves of these free DNA samples showed qPCR products displaying a single peak with non‐specific bands, indicating the absence of the *EGFR* gene exon 19 D15 type mutation.

**FIGURE 12:**
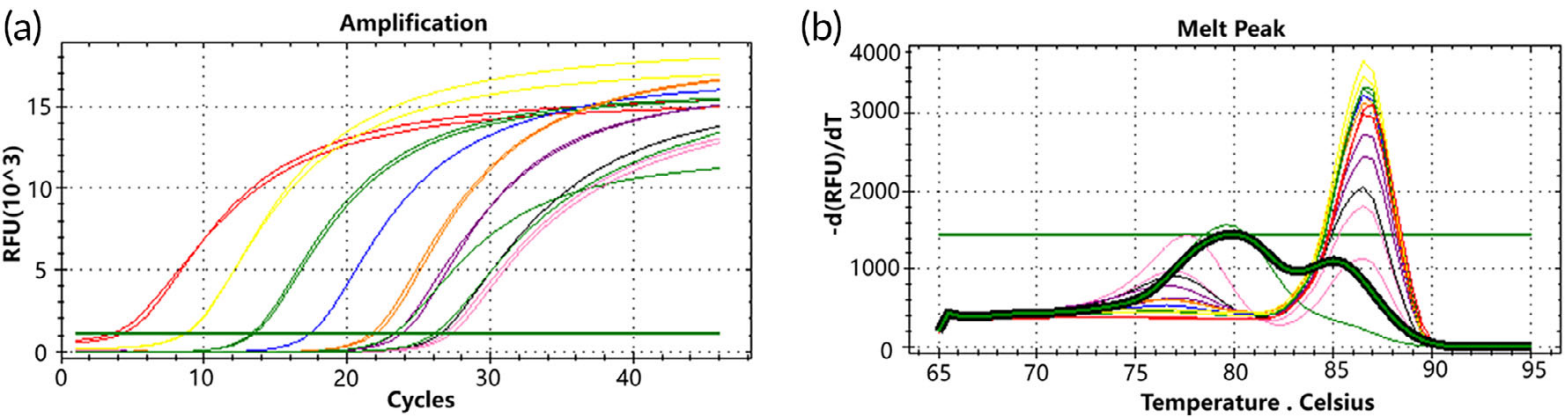
qPCR amplification curve and melting curve of D15 mutant molecular switches in negative control.

Figure 12 depicts the qPCR amplification and melting curves of the D15 mutant molecular switch in the negative control. These curves were comparable to the amplification and melting curves of the free DNA samples with the detected D15‐type mutation. This similarity suggested that the D15 mutant molecular switch was highly sensitive but might produce false‐positive results. Further optimization was necessary to increase the specificity of its detection in clinical free DNA samples.

**FIGURE 13:**
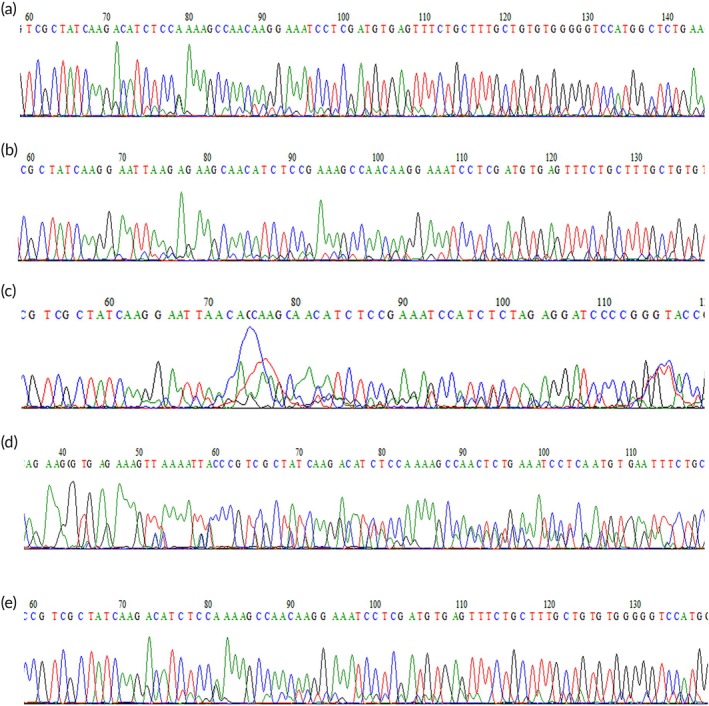
gDNA *EGFR* gene exon 19 sequencing profiles of lung cancer tissues from 5 patients with corresponding free DNA samples.

Out of the 24 DNA samples, the molecular switch technique detected the D15‐type mutation in 5 samples. The corresponding lung cancer tissue samples were sequenced, and all 5 were found to have the D15‐type mutation in exon 19 of the *EGFR* gene. These samples exhibited heterozygosity for the coexistence of the wild‐type sequence and the mutated sequence (Figure 13).

Validation using sequencing of corresponding lung cancer tissue samples from 24 patients with free DNA confirmed the presence of the *EGFR* gene D15‐type mutation in 8 tissue samples. Therefore, the concordance of the molecular switch technology for detecting the *EGFR* gene in blood free DNA and tumor tissues of patients with lung cancer in this study was 62.5%.

## 4 DISCUSSION

Direct sequencing and Amplification Refractory Mutation System (ARMS) methods are commonly used for *EGFR* mutation detection in clinical and scientific research.^26^, ^27^, ^28^ However, direct sequencing has limited detection capability, with a sensitivity of about 10%, and the steps are complex, time‐consuming, and labor‐intensive. The ARMS method utilizes mismatched bases to inhibit the catalytic reaction of DNA polymerase. When the template and primer are not paired, amplification of the template DNA is prevented.^29^ The ARMS method has offered advantages due to its simplicity, rapidity, high sensitivity, and high specificity. However, it requires that the 3′ terminal bases of primers be allele‐specific, and the experimental design and operating conditions are more stringent.^30^, ^31^

The molecular switch assay developed in this study is akin to the ARMS method, but integrates sulfide‐modified bases to enhance the inhibition of high‐fidelity DNA polymerases at mismatched bases, thereby improving the specificity of allele‐specific PCR. This molecular switch assay utilizes high‐fidelity DNA polymerase with sulfide‐modified specific primers, enabling the detection of *EGFR* gene exon 19 base 15 and base 18 deletion mutations on a Real‐time qPCR platform with heightened sensitivity and specificity. In this study, the D15 mutant molecular switch demonstrated superior sensitivity and specificity compared to the D18 variant. Moreover, assay reliability can be bolstered through additional manipulations, such as increasing the number of parallel tube repeats in the D18 mutant molecular switch. Blocker primers, a proposed design protocol aimed at reducing false‐positive amplification in high‐sensitivity qPCR methods, have been extensively applied in various PCR probes or systems.^24^, ^25^, ^32^, ^33^ Statistical comparison of the standard curve indicated that when used in conjunction with the molecular switch method, Blocker primers more effectively detected mutations in mutant templates amid a low‐copy genomic DNA background compared to detecting mutations in a low‐copy template background without Blocker primers. This underscores their suitability for detecting samples with lower template and mutation copy numbers.

As symptoms of lung cancer are often not immediately apparent, 80% of patients are typically diagnosed in the middle to late stages of the disease. Early screening for lung cancer is crucial for detecting tumors and providing timely treatment, which can improve patient survival rates. Common methods for early lung cancer screening include imaging, tumor marker detection, and tissue biopsy. However, imaging tests can pose risks due to radiation exposure, and lung cancer marker detection has limitations such as low sensitivity and specificity, leading to high rates of false positives and false negatives. Tissue biopsy, considered the gold standard for diagnosing lung cancer, is invasive and offers limited opportunities for repeat sampling. Thus, current methods for early lung cancer screening are inadequate for widespread screening of asymptomatic individuals. In recent years, liquid biopsy techniques using free DNA samples have gained popularity for early lung cancer screening.^34^, ^35^, ^36^ This study assessed the feasibility of the molecular switch assay in detecting *EGFR* exon 19 D15 type mutations in free DNA samples. The consistency of mutation detection in low‐copy free DNA samples was determined to be 62.5%. Possible factors influencing detection variability include variations in clinical stages among selected patients and the progression of the disease.

Expanding the target sites for *EGFR* gene mutation detection can help align the results of mutation detection in free DNA from blood with the actual mutation spectrum. Additionally, the amount of free DNA is closely associated with the stage of the primary tumor. For example, higher frequencies of *EGFR* gene mutations may be linked to larger primary tumors in patients with advanced NSCLC. These tumors may also exhibit a greater propensity for distant metastasis, resulting in increased release of tumor cells into the bloodstream. Consequently, this leads to higher concentrations of mutant DNA in the blood and enhances the likelihood of detecting mutations. Moreover, the gene mutation detection technology discussed in this context can improve the detection rate of mutations present in low‐copy numbers by enriching the targeted region in the sample template. For instance, the immunomagnetic bead method can enrich the mutated region of the *EGFR* gene, highlighting the strong compatibility of molecular switch assay in mutation detection.

## 5 CONCLUSIONS

In this study, we assessed the sensitivity and specificity of molecular switch technology combined with blocker primers for detecting *EGFR* exon 19 mutations. We demonstrated that this novel method allows real‐time detection of mutated templates on a qPCR platform. The use of molecular switch assay in conjunction with Blocker primers enhances the detection sensitivity of free DNA samples with lower copy numbers. This advancement holds promise for improving early lung cancer diagnosis, facilitating efficient non‐invasive detection methods, and enabling personalized treatment strategies.

## AUTHOR CONTRIBUTIONS

**Li Xiao:** Funding acquisition; writing – review and editing; project administration; resources.

## CONFLICT OF INTEREST STATEMENT

The authors declare no conflicts of interest.

## Data Availability

The data that support the findings of this study are available on request from the corresponding author. The data are not publicly available due to privacy or ethical restrictions.
